# Arthroscopic arthrodesis for ankle arthritis without bone graft

**DOI:** 10.1186/s13018-016-0490-y

**Published:** 2016-12-01

**Authors:** Xiaojun Duan, Liu Yang, Li Yin

**Affiliations:** Center for Joint Surgery, Southwest Hospital, The Third Military Medical University, 30 Gaotanyan St., Shapingba, Chongqing, 400038 China

**Keywords:** Ankle, Arthritis, Arthrodesis, Arthroscopy, Treatment

## Abstract

**Background:**

Ankle arthrodesis is considered by many to be the standard operative treatment for end-stage ankle arthritis. The purpose of this study was to perform a new technique for ankle joint surface and determine the outcome for the union rates of ankle arthroscopic arthrodesis.

**Methods:**

A total of 68 patients with posttraumatic arthritis, primary osteoarthritis, and rheumatoid arthritis were treated by ankle arthroscopic arthrodesis between May 2007 and December 2012. Our surgical indication was deformity less than 15° measured by weight-bearing radiographs. Firstly, the remaining articular cartilage was removed with different curettes and shavers. Then, the new technique (microfracture) was done at tibiotalar surfaces. Finally, the ankle was fixed with two cannulated percutaneous screws. The wound healing, complications, postoperative radiographs, and American Orthopaedic Foot and Ankle Society (AOFAS) score were evaluated.

**Results:**

The average follow-up time was 32 months (range 25–58 months). There was no bone grafting, and a fusion rate of 100% was achieved. The average fusion time was 12.1 weeks. One patient developed superficial infection at 2 weeks postoperatively and was cured by nonsurgical treatment. No deep infections, deep venous thrombosis, or revision surgery were observed. Screws had been removed in four patients because of prominence. One patient had fusion in the subtalar joint because of arthritis at 5 years postoperatively. At the last follow-up, radiographic signs of developed or progressing arthritis were observed in nine patients at subtalar joint and in four patients at talonavicular joint. At 1-year follow-up, the mean AOFAS ankle/hindfoot score had increased to 84 from a mean preoperative value of 38 (*P* < 0.01).

**Conclusions:**

Arthroscopic arthrodesis provides surgeons with an alternative to traditional open techniques for the management of severe ankle arthritis. Our data show that preparation of the joint surface with microfracture is an effective technique to increase the union rate of arthroscopic ankle arthrodesis, while bone graft and other promoting substances are not necessary to be routinely used.

**Electronic supplementary material:**

The online version of this article (doi:10.1186/s13018-016-0490-y) contains supplementary material, which is available to authorized users.

## Background

Ankle arthrodesis can result in a painless, normal walking gait for patients with end-stage ankle arthritis. It should be considered after the failure of conservative treatments [1–4]. Since the first arthrodesis performed in the early ninetieth century, technological advancements and better understanding of the ankle anatomy have brought about less-invasive surgical procedures. Upon these improvements, many surgical techniques from external fixation to internal fixation have been developed, in order to obtain smaller invasion, fewer complications, and better outcomes. Symbolically, Schneider performed the first arthroscopic ankle arthrodesis [5].

So far, arthroscopic ankle arthrodesis has gained in popularity due to increased experience and improved instrumentation [3]. The fusion rate between arthroscopic and open arthrodeses is comparable, but the arthroscopic method has been deemed to have shorter union time, less blood loss, less morbidity, shorter hospital stays, and more rapid mobilization [6–8]. Despite these advantages, some concerns have been expressed regarding arthroscopic ankle fusion, including the ability of correcting significant angular deformities, bone loss, and others with the arthroscopic technique [9–11].

The goal of the current retrospective study was to evaluate the application of a new technique for ankle joint surface and to analyze whether it can increase the union rate of ankle arthroscopic arthrodesis or not. The results would also be compared with other similar techniques.

## Methods

### Patient population

Inclusion criteria: patients with less than 10° of deformity in the coronal plane before 2009 were chosen; yet literature review [9, 12] and our preliminary results led us to subsequently change the surgical indications to less than 15° of deformity; the primary diagnosis was posttraumatic arthritis, primary osteoarthritis, or rheumatoid arthritis. Exclusion criteria: concomitant diseases, including subtalar arthritis or talar necrosis, were excluded because the combined surgery was needed; stiff ankle were excluded because the space for arthroscopy was too limited; the primary diagnosis of tuberculous arthritis, active infection, Charcot’s disease, or tumor was excluded because various factors could influence prognosis.

Unilateral arthroscopic ankle arthrodeses were performed in the 68 patients (38 males and 30 females) by two senior surgeons between May 2007 and December 2012. The average age of the patients was 59 years (range 30–83 years). All patients received two cannulated, compression percutaneous screws (INTEGRA, International Ltd, France). Thirty-five of the 68 patients (51.5%) had posttraumatic arthritis, 24 had primary osteoarthritis (35.3%), and 9 had rheumatoid arthritis (13.2%). Consents had been obtained from patients who permitted the use of individual data for research and publication.

Prior to any operative measurements, patients were asked to complete an ankle-hindfoot questionnaire, which was developed by the American Orthopaedic Foot and Ankle Society (AOFAS) as a standard method to assess the clinical status of ankle-hindfoot (Table 1) [13]. The scale incorporated both subjective factors from the patients’ questionnaire (e.g., pain and activity limitations) and objective factors from the surgeons’ questionnaire (e.g., gait abnormality and alignment). The same questionnaires were repeated at 1-year follow-up.

**Table 1:** The table shows the American Orthopaedic Foot and Ankle Society (AOFAS) ankle-hindfoot scoring system that was used in the current study. Patients were given this questionnaire preoperatively and postoperatively to evaluate subjective and objective outcomes

Parameter	Points
Pain (40 points)	
None	40
Mild	30
Moderate	20
Severe	0
Function (50 points)	
Activity limitations	
None	10
Limitations on recreational activities	7
Some limitations on daily and recreational activities	4
Severe limitations on daily and recreational activities	0
Maximum continuous walking distance	
600 m or more	5
400 m to less than 600 m	4
100 m to less than 400 m	2
Less than 100 m	0
Walking surfaces	
No difficulty on any surface	5
Some difficulty on uneven terrain, stairs, and inclines	3
Severe difficulty or inability to walk on uneven terrain, stairs, and inclines	0
Gait abnormality	
None or slight	8
Obvious (walking possible but gait abnormality obvious)	4
Marked (walking difficult and gait abnormality obvious)	0
Sagittal motion (flexion plus extension)	
Normal or mild restriction (30° or more)	8
Moderate restriction (15°–29°)	4
Severe restriction (less than 15°)	0
Hindfoot motion (inversion plus eversion)	
Normal or mild restriction (75–100% normal)	6
Moderate restriction (25–74% normal)	3
Severe restriction (less than 25% normal)	0
Ankle-hindfoot stability (anterior drawer, varus-valgus stress)	
Stable	8
Unstable	0
Alignment (10 points)	
Good, plantigrade foot, well aligned	10
Fair, plantigrade foot, mild to moderate degree of malalignment	5
Poor, nonplantigrade foot, severe malalignment	0

### Preoperative evaluation

As previously described, preoperative work-up for ankle arthrodesis should take into account several factors, such as axial deformities, bone defects, bone quality, condition of the skin, and underlying infections [10]. Inspection of tibiotalar joint usually revealed reduced, sometimes almost abolished range of motion; pain and swelling were common as well. During physical examination of the arthritic ankle, it was important to evaluate adjacent joints. These joints (knee, subtalar, and tarsal) would be needed to compensate for the motion loss due to ankle fusion and should be free of degenerative changes [14, 15]. Weight-bearing radiographs of anteroposterior, lateral, and mortise views of the ankle were required. The rearfoot alignment (Cobey/Saltzman) view was also essential to evaluate the ankle joint and to identify any calcaneal-to-tibial deformities [16]. In the coronal plane, the lateral distal tibial angle (LDTA), the tibiotalar angle, and the calcaneal tibial alignment should be measured [17]. MRI and CT scans were useful when evaluating bone defects (for example, necrosis of the talus and pilon fracture) and pathologies involving soft tissues [10].

### Surgical technique

The patient was placed supine under general or spinal anesthesia. Preoperative intravenous antibiotic prophylaxis was performed (usually the first generation cephalosporin). A suitably sized sandbag under ipsilateral buttock was used to maintain the position of the limb. The thigh was supported by a well-padded holder attached via a clamp to the rail of the table. A tourniquet was positioned around the thigh and inflated (systolic blood pressure + 100 mmHg, usually about 270 mmHg). A bump was positioned under the thigh. The leg was prepared up to the knee. It was necessary to prepare the leg high enough to assess limb alignment and to have good access to place guidewires and screws for fixation. Fluoroscopy must be ready to use. In most cases, a slight noninvasive traction could be applied to the foot for better visualization of the joint [18–20].

**Fig. 1:**
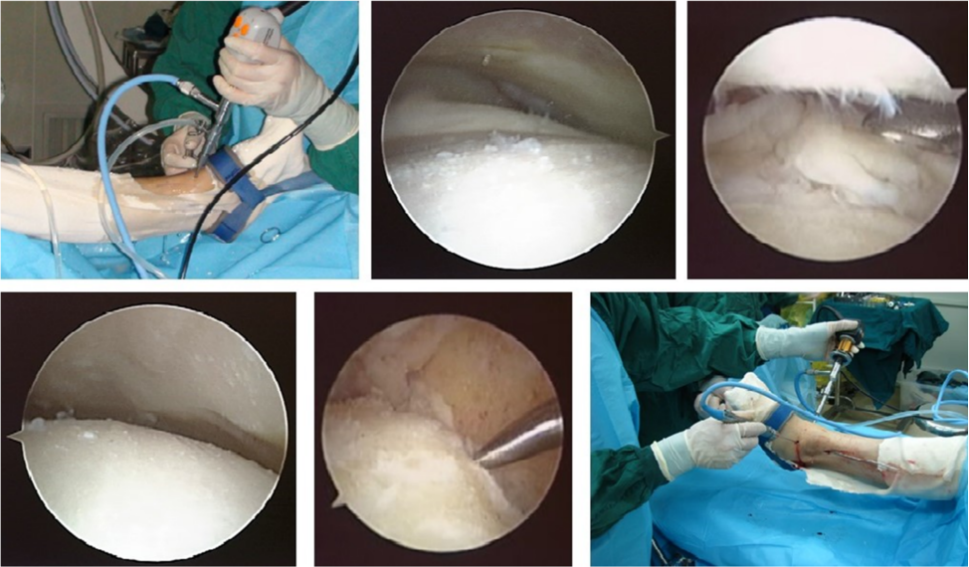
Preparation of the articular surface


Arthroscopy was performed with a 2.7- or 4.0-mm 30° arthroscope. Before the two standard portals (anteromedial and anterolateral) were established, the joint was injected with 20 mL of saline solution in order to expand the joint space. The anteromedial portal (medial to the tibialis anterior tendon) was placed first. The anterolateral portal (lateral to the extensor digitorum communis tendon) was established under direct vision. When creating the anterolateral portal, attention should be paid not to damage the superficial peroneal nerve. In skinny patients, this can be appreciated in the subcutaneous tissue preoperatively (with the foot inverted and the toes flexed) and marked with a surgical pen. Both portals were performed with a skin incision and a blunt dissection of the subcutaneous tissue with a mosquito clamp or a trocar. After the portals been established, debridement of the soft tissues was performed with a shaver in the anterior part of the joint. Once adequate visualization had been achieved, the posterolateral portals were established for fluid flow. The entire cartilage was removed with different curettes, shaver, and an acromioplasty bur. In some cases, resection of anterior tibiotalar osteophytes was required to access the joint better. The lateral malleolus articular surface was removed as well. Thereafter, microfracture was performed at both of the tibiotalar surfaces (Fig. 1, Additional file 1: Video S1). The instruments were similar to that in the treatment of osteochondral lesions of the talus. The tourniquet could be let down in order to evaluate bleeding from the tibial and talar surfaces.

Additional file 1: Video S1. Arthroscopic view after joint surface preparation. (WMV 1975 kb)


Once accurate preparation had been completed with adequate bleeding observed, two guiding pins were drilled percutaneously into the tibial plafond from medial and lateral sides under direct vision. The tips of the pins should be examined carefully to predict corresponding penetration points in the talar dome. Then, the traction was released and the ankle was realigned. The foot and ankle were held in neutral dorsiflexion, with 0° to 5° hindfoot valgus and external rotation equal to the opposite side. If the opposite side was abnormal, the operated ankle was then positioned at 5° to 10° of external rotation [6, 12]. While this position was maintained, the guide pins should be advanced into the talus. The position and depth of the pins should be determined using fluoroscopy. Fixation was achieved with internal fixation. Two cannulated, interfragmental compression percutaneous screws (usually the diameter of 7.5 mm) were placed under image intensifier control. Position of the screws might vary according to the surgeon’s preference. Crossed transverse configuration, as well as a parallel—almost longitudinal—positioning of the screws, must lead to a satisfactory primary stability. The incisions were then closed with simple sutures. A single drainage was performed before 2009. Literature review and our preliminary results led us to subsequently waive the drainage.

### Postoperative care

After surgery, a complete below-knee cast was applied, and the patient was kept non-weight bearing for 6 weeks. Then, a removable boot was applied, and the patient was allowed partial weight bearing for 4–6 weeks. At 12 weeks after surgery, if clinical and radiological signs of fusion were present, the patient could return to full daily activities.

## Results

**Fig. 2:**
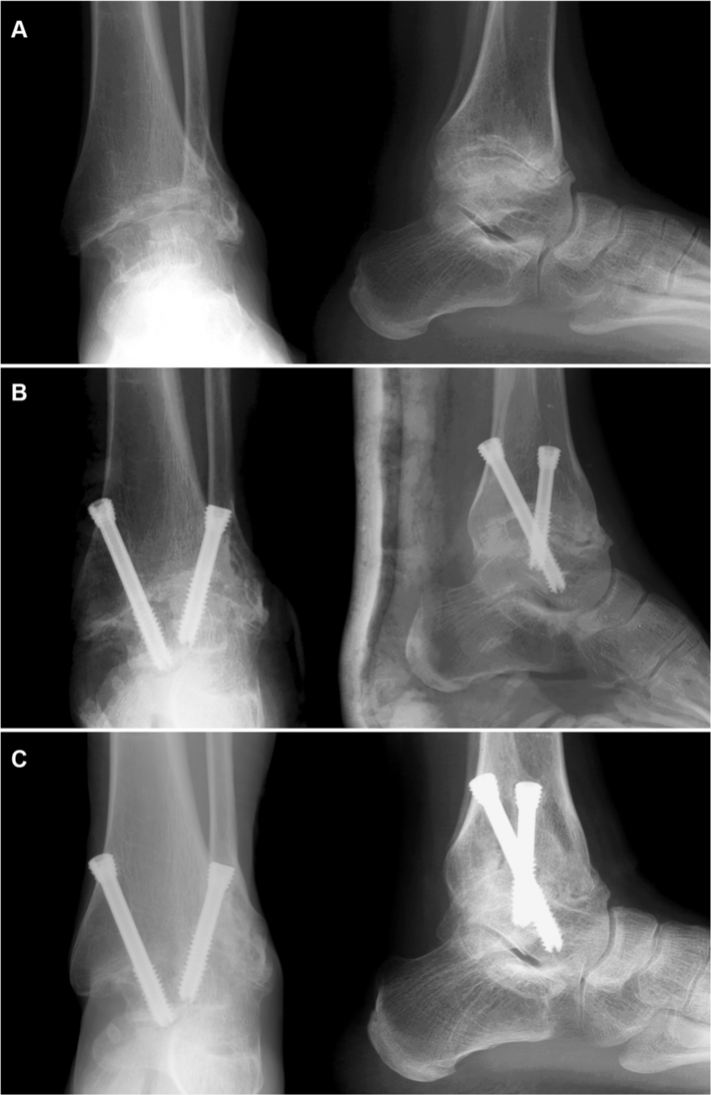
Case 1. **a** Preoperative radiographs. **b** Radiographs at 1 day postoperatively. **c** Radiographs at 4 years postoperatively

**Fig. 3:**
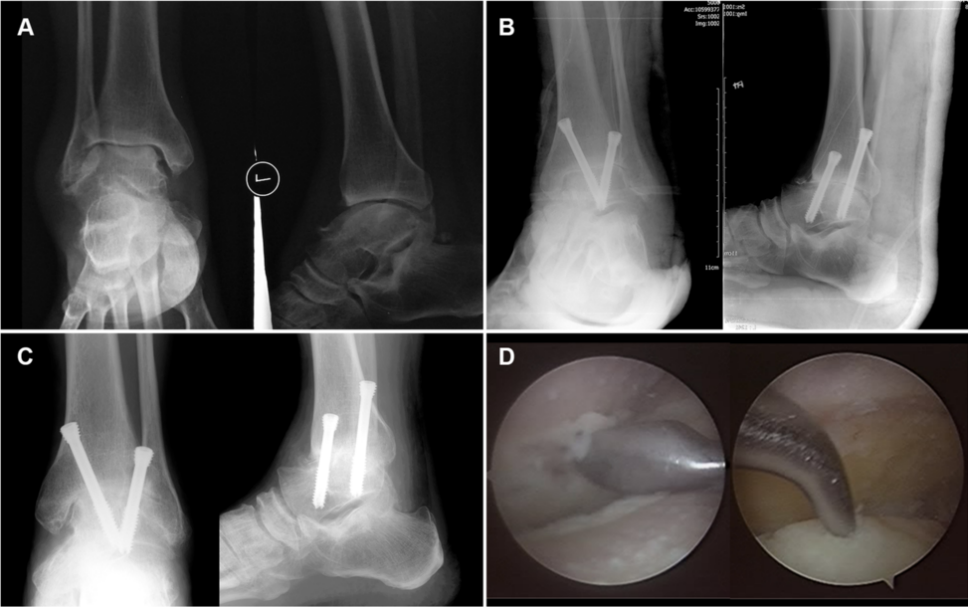
Case 2. **a** Preoperative radiographs. **b** Radiographs at 1 day postoperatively. **c** Radiographs at 12 days postoperatively. **d** Arthroscopic surgery in progress


The average follow-up time was 32 months (range 25–58 months). There was no bone grafting, and a fusion rate of 100% was achieved (Fig. 2, Additional file 2: Video S2 and Fig. 3, Additional file 3: Video S3). The average fusion time was 12.1 weeks. One patient (1.5%) developed superficial infection at 2 weeks postoperatively. The inflammation was settled with dressing changes and a short course of antibiotics. No deep infections, deep venous thrombosis, or revision surgery due to malalignment were observed.

Additional file 2: Video S2. Postoperative functional evaluation of a female patient who underwent arthroscopic arthrodesis for left ankle arthritis (case 1). (WMV 3794 kb)



Additional file 3: Video 3S. Postoperative gait of a male patient who underwent arthroscopic arthrodesis for left ankle arthritis (case 2). (WMV 7552 kb)


Screws were removed in 4 patients at 1–2 years postoperatively because of prominence. At the last follow-up, radiographic signs of developed or progressing arthritis were observed in 9 patients (13%) at the subtalar joint and in 4 of 68 patients (6%) at the talonavicular joint. One patient had subtalar fusion at 5 years postoperatively because of arthritis and varus malalignment in the subtalar joint. The other patients declined further surgery because they were relatively pain free after nonoperative treatments.

At 1-year follow-up, the mean AOFAS ankle/hindfoot score had increased to 84 from a mean preoperative value of 38 (*P* < 0.01).

## Discussion

In this study, we aimed to evaluate the outcomes of a new technique in ankle arthrodesis. Sixty-eight patients who underwent ankle arthroscopic arthrodesis with microfracture technique were retrospectively investigated with an average follow-up time of 32 months. Satisfactory results of a union rate of 100% were achieved.

In the last two decades, the popularity of this technique has been increasing due to the advantages mentioned above [3]. Best MJ et al. reported that from 1994 to 2006, the population-adjusted rates of foot and ankle arthrodeses increased by 146%. The number of outpatient arthrodeses performed with arthroscopic assistance increased by 858% [21]. The capability of treating ankles with marked deformity, slightly shorter time of union, reduced complication rates, and lower costs compared with open surgery make arthroscopic ankle fusion a safe and reliable technique [10, 12, 22–25].

### Preparation of the articular surface

It is very important to prepare the articular surface for ankle fusion [1, 2]. Most reports of arthroscopic ankle arthrodesis have recommended preparation of both tibial and fibular articular surfaces. Ferkel et al. reported the procedure: the entire articular surface of the tibial plafond, talar dome, and medial and lateral talomalleolar surfaces should be systematically removed [26]. Zwipp H et al. reported a generous debridement, as well as the medial and lateral gutters [27]. But Elmlund AO and Winson IG recommended that the remaining articular cartilage could be removed with a combination of a 4.5-mm soft-tissue debrider and curettes [12, 20]. The medial malleolar articular surfaces are removed, but the lateral gutter is only cleared enough to allow compression of the joint or reduction of deformity, and the articular surfaces are not addressed. Our experience is that the entire articular surfaces, including the lateral gutter, should be removed. It is difficult to observe the solid union sometimes because the joint gap is very narrow at the tibiotalar joint and medial gutter after compression, while the sign of callus at the lateral gutter can be easily observed when solid union is achieved.

To facilitate bleeding, 1 mm of bone is slightly abraded with the bur. Ferkel et al. preferred to use the bur to create multiple small dimples, or spot welds, onto the surface of the tibia and talus to facilitate early bony union [26]. Winson IG et al. reported using a bur to remove the bone down to healthy cancellous bone [12]. Zwipp H et al. preferred to remove all sclerotic and nonviable bone [27]. Sometimes, the gap appears obviously after a lot of sclerotic bone has been removed and it may affect union. Many patients requiring ankle arthrodesis have a significant degree of limb-length discrepancy as a result of severe bone loss. Therefore, we strictly control the removal of the bone after thorough removal of the articular cartilage to prevent further bone loss. We only abrade the sclerotic bone gently to the underlying subchondral bone for fresh. The technique of microfracture will be applied at both of the tibiotalar joint surfaces. The bone marrow and mesenchymal stem cell will fill the gap to facilitate early bony union [28, 29]. It is the similar principle when the surgeons use a 1- or 2-mm Kirschner wire to drill into the subchondral bone to prepare the joint for open arthrodesis [30]. The advantages of this technique include avoiding excessive bone loss at the arthrodesis site, decreasing the limb-length discrepancy, and maintaining the surface profile.

### Screw fixation

Relative to external fixation, internal fixation may provide earlier fusion and higher fusion rates, a greater degree of patient satisfaction and decreased complications, especially soft tissue infections [31]. There are over 40 techniques documented in the literature, such as open-crossed screw constructs and plates, intramedullary nails (IMNs), and external fixation devices [32, 33]. But for arthroscopic arthrodesis, only screws are chosen.

Ferkel et al. reported two cannulated screws for fixation, one inserted from medial malleoli and the other one inserted from lateral malleoli [19, 26]. Both screws originated at the posterior aspect of the malleoli and were orientated 30° inferiorly and 30° anteriorly. Winson IG et al. reported that two cannulated percutaneous ACE 6.5-mm screws were placed medially from the tibia into the talus under image intensifier control and were kept parallel on both AP and lateral views [12]. Zwipp H et al. reported that the arthrodesis was fixed with four 6.5-mm cancellous lag screws [27]. Two screws were inserted parallel from the anterior aspect of distal tibia into the body of talus. The third screw, which was mechanically most important, was inserted through a posteromedial stab incision (approximately 3 cm proximal to the tip of the medial malleolus) and into the anterolateral portion of the talar head. The fourth screw was inserted percutaneously from the posterolateral aspect of distal fibula (approximately 1.5 cm proximal to the tip of lateral malleolus) into the dorsal portion of the talar body. A study led by Yoshimura I et al. showed that arthroscopic ankle arthrodesis achieved a high rate of union, with the fastest union achieved with three parallel screws placed medially from distal tibia into talus. Other screw configurations used in this study included three transmedial and translateral malleolar screws, two transmedial and translateral malleolar screws, and two transmedial malleolar screws [34].

Obtaining rigid fixation has the absolute priority, while understanding compression is critical to ankle arthrodesis. The surface of arthrodesis would be decreased when too many screws cross the joint. In our practice, two crossed screws are strong enough to stabilize the fixation. The screw from the posteromedial malleolus to the anterior talus can produce compression; the other from the lateral tibia maintains the strength of anti-rotation. Anatomical structures at risk include the dorsalis pedis artery and deep peroneal nerve, which locate in front of the joint capsule. The safe approach for lateral guide pin is across the anterior fibula and lateral tibia to the center of joint. If bone defect exists across the paths of screws, the direction of screws should be adjusted.

### Bone graft

Since union is the primary goal of ankle fusion, nonunion should be considered as the main undesirable complication. The use of bone graft with internal or external compression will enhance the likelihood of a successful arthrodesis [31, 35–37]. In studies ranging in size from 12 to 101 patients, rates of successful primary ankle fusion of 80–100% have been reported. Up-to-date arthroscopic fusion reported a nonunion rate ranging from 3 to 15% [12, 18, 22, 38, 39]. Commonly reported risk factors for nonunion are poor bone quality, massive bone defect, and inherent positional ankle deformity [6, 38]. No advantages have been shown by the addition of demineralized bone matrix or platelet-rich plasma [38].

Mohamedean A et al. reported that a rate of open ankle fusion of 100% was achieved at an average of 12.2 weeks, while an iliac bone graft was used in two of their patients with old pilon fractures [40]. Zwipp H et al. reported that union occurred in 93 of 94 patients (99%) and 38 of 94 cases were grafted with autologous bone [27]. A monocortical bone block was taken from the ipsilateral iliac crest, and additional cancellous bone chips were interposed. Myerson M et al. thought that it was not necessary to use bone graft when the bone defect was smaller than one-third surface [41]. In the current study, the deformity was limited and the bone defect was not huge. We used the technique of microfracture to facilitate early bony union. So our routine surgical technique did not include bone graft and other demineralized bone matrix.

The main limitation of this study is its small sample size. This can be improved by multi-center study in the future.

## Conclusions

Arthroscopic ankle arthrodesis is a good option for end-stage ankle arthritis. The new technique involves microfracture being done after thorough removal of the articular cartilage, which benefits to bone union. Two crossed screws can maintain rigid fixation. Our results are similar to those of open and arthroscopic ankle arthrodesis, yet with lower complication rates and higher fusion rate.

## Abbreviations

AOFAS: American Orthopaedic Foot and Ankle Society
IMNs: Intramedullary nails
LDTA: Lateral distal tibial angle
